# Research on the correlation between component elements and psychological perception of the spatial form of underground commercial street corners based on virtual reality technology

**DOI:** 10.3389/fpsyg.2022.950593

**Published:** 2022-09-06

**Authors:** Liang Sun, Tianyu Fang, Yan Sun, Bo Wang, Zebiao Shao

**Affiliations:** School of Architecture and Design, China University of Mining and Technology, Xuzhou, China

**Keywords:** virtual reality technology, underground commercial street, corner, spatial form, component elements, psychological perception

## Abstract

The corner space, an important area of underground commercial streets, not only converts space functions but also exerts a great impact on the space atmosphere by transforming the environmental quality of the commercial street space. Based on corner space investigations of several underground commercial streets in China, this paper constructs a realistic scene model using virtual reality technology and screens. This paper classifies the corner space elements of underground commercial streets through preliminary experiments. Based on the conclusions, different morphological models of the corner space were constructed by orthogonal experiments and virtual reality technology combined with psychology. A semantic differential was used to quantitatively evaluate and analyze the spatial experience cognition of the subjects. This enabled an analysis of the of underground commercial street corners’ relationship to the component elements and the visitors’ psychological perception of their spatial form.

## Introduction

With the continual urbanization of China, the urban population has increased dramatically, and problems such as traffic congestion and land shortages are becoming prominent. Therefore, developing urban underground space has become a realistic method to deal with the “metropolitan malaise.” (Tong, 2005) This diversified urban space expansion on underground commercial streets is used mainly by those seeking food and entertainment, causing various corner spaces to be utilized. Corner spaces have become an important area of commercial streets. They not only convert space functions, but they can also stimulate their visitors’ psychological perceptions, thereby affecting the atmosphere and environmental quality of the underground space. However, at present, the design of corner space has not received proper attention. Some street corners are simple in design and complicated in function, which means they cannot positively stimulate people’s psychological perception. This affects the commercial vitality and value of underground space to a certain extent. Therefore, how to reshape the form of the corner space to improve the overall quality of the underground environment is a factor that architects need to focus on when designing underground space.

Urban underground space, with spatial and social attributes, produces and ultimately affects social behavior and cognition in urban social relations. It has become a research hotspot in the fields of architecture and urban planning. In the early 1980s, extensive research was conducted on the psychology, cognition and evaluation of the underground space environment. In Wada and Sakugawa (1990) studied the psychological and physiological effects of underground space and gave high credibility to the simulation of underground space as part of their research. In Carmody and Sterling (1993) determined that the cause of people’s cognitive response (especially negative response) to underground space is closely related to the physical properties of underground space. In Bartel and Janssen (2016) proposed that the main problem of sustainable development of underground space in the future is to solve the contradiction between the current rapid development of underground space and the increasing psychological demands of people. In Yao et al. (2021) developed an ideal underground shopping street using 3dmax software to conduct space modeling and lighting simulation experimenting with changes in width, height and other independent variables thereby setting up several models before importing the final model into Vizard 5 for script editing to generate an ideal VR space for an underground commercial street. Forty-seven people participated in 80 groups of experiments to determine the best 3D walking space. Mao et al. (2020) proposed that the influence of underground space environment on people’s behavior and psychology is important when evaluating the quality of urban underground space. Li et al. (2021) used the method of electroencephalogram (EEG) + virtual reality (VR) + laboratory environment control (LEC) to simulate the underground building environment and revealed the law of interaction between building space information and human perception feedback. Sun et al. (2020) adopted digital means, such as VR, eye trackers and multi-channel sensors, combined with methods like a questionnaire survey, SD, and behavior mapping, to study the environmental characteristics and a perception evaluation of underground space. With the progression of research on underground space environment and human perception, the interdisciplinary exploration mode has gradually taken shape, and advanced technologies such as VR have been applied to the research process. However, current research focuses mainly on the influence of underground space environment on human behavior and psychology on a macro level. This approach lacks the micro-level exploration on the correlation between the architectural form elements and psychological perception. The research depth and breadth also need to be further expanded.

This paper presents the corner space of the urban underground commercial street as the research object. The authors conducted variable control experiments by using a virtual reality technology experimental platform with on-site investigations, field measurements, observations, and analyses. The authors quantitatively measured and systematically analyzed the correlation between the components and psychological perception of an underground commercial street’ by using physiological technology such as eye movement analysis and multi-channel physiological sensors as well as the semantic differential method to measure subjective responses or attitudes. All of these tests were used to design user-friendly corner spaces in underground commercial streets. This paper also provides a theoretical basis for design optimization and performance evaluations for architects.

## Research design

**Figure 1:**
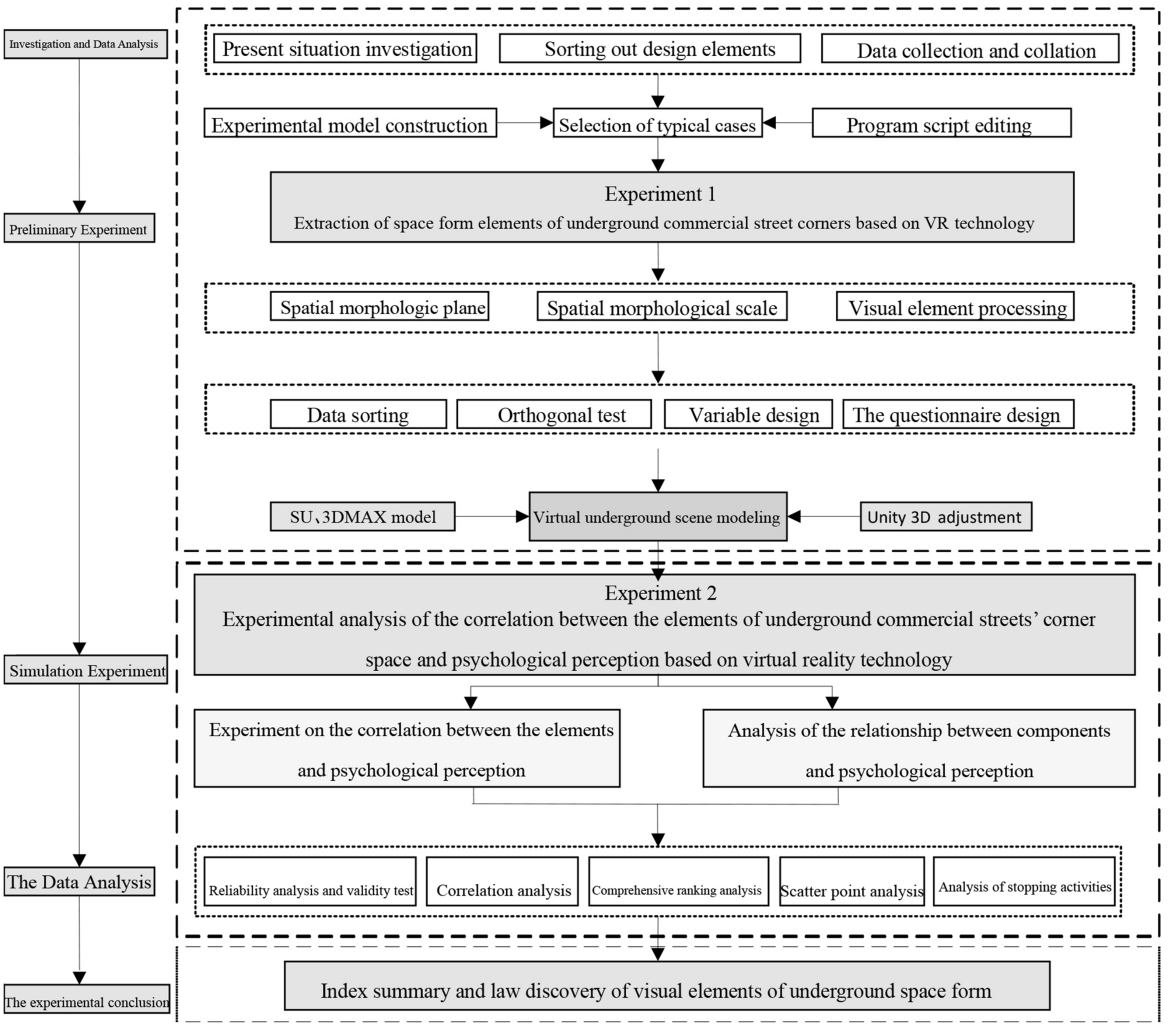
The research technology route.

To investigate the correlation between the constituent elements of the corner space of an underground commercial street and the psychological perception, this research is carried out in four steps: (1) investigation and data analysis, (2) preliminary experiment, (3) simulation experiment, and (4) analysis and discussion of experimental data. This research contains two experiments: the pre-experiment and the simulation experiment. The main purpose the first preliminary experiment (pre-experiment) is to extract the constituent elements the underground commercial street’s corner space. The second simulation experiment aims to analyze the correlation between the corner space elements and the psychological perception based on the conclusions of the pre-experiment. The research technology route is shown in Figure 1. The four research steps can be summarized as follows:

1. **Investigation and Data Analysis.** For the investigation of underground commercial street intersections and corner spaces, basic data were collected. The constitutive elements of the underground commercial street corner spaces were sorted out according to architectural typology theory. On this basis, the interface types of underground spaces were classified and recorded.
2. **Preliminary Experiment.** Based on the research samples of corner space and their classification, a typical scene model was established of the corner space for an underground commercial street. A preliminary experiment was performed to simulate typical scenes with virtual reality technology. The main elements of the underground space were extracted by analyzing the constituent elements of corner space and their effects on the underground space experience. The authors also verified the reliability of virtual reality technology by simulating the spatial form of an underground commercial streets, which provided a scientific basis for a later simulation experiment.
3. **Simulation Experiment.** A virtual scene model of the underground commercial street corner space was established based on the constituent elements of the underground space extracted from the pre-experiment. With the virtual scene model simulated by virtual reality technology, as well as experimental psychology and semantic analysis, the cognitive evaluation of the subjects’ spatial experience was quantitatively analyzed. On this basis, the relationship was investigated between the underground commercial street’s elements of corner space and the viewer’s psychological perception.
4. **Analysis and Discussion of Experimental Data.** The quantitative data were analyzed and processed by professional statistical software. Moreover, the relationship between the constituent elements of the underground commercial street corner space and the psychological perceptions of users were explored through correlation analysis, factor analysis and scatter diagram analysis. Based on the obtained relationship, some suggestions were put forward on the corner space design of underground commercial streets.

## Extraction of space form elements of underground commercial street corners based on VR technology

To explore the space form elements of underground commercial street corners, this section analyzes the relationship between corner space form and its elements. The objective is to clarify the importance and classification of the elements through investigative data and experiments, thereby laying a foundation for further research.

### Research design and data collation

This study focuses on the shape and components of the corner space of underground commercial streets. In order to clearly show the constituent elements, we obtained a collection of basic data that identifies the types of each interface on urban underground commercial streets. The research objects are the intersection and corner space of these streets. The research content mainly includes quantitative design elements of their planar shape, length, width, height, and spatial processing, as well as the height and width of shop doors. The method adopted is field research with the use of an electronic map to increase the richness and coverage of selected samples showing planar and spatial dimensions.

The specific steps of investigation and data collation are as follows. The first step was to conduct an on-the-spot investigation of underground commercial streets in Shanghai, Nanjing, Chengdu, Zhengzhou, and Xuzhou. In this investigation, the corner space was photographed from multiple angles, and the length, width, height and inside storefront dimensions were measured by a VCHON High Precision 60 M Laser Range Finder (Electromann SA, Johannesburg, South Africa). The corner angle data were measured by planar drawings. The second step was to improve the field survey data. With the comprehensiveness and accuracy of electronic map information, the survey data was corrected and improved to ensure its authenticity and reliability. The third step was to sort out and summarize the survey data. We sorted out the planar shape, space shape and scale data of the samples, as well as the frequency and proportion of their appearance in the samples, so as to provide data support for the establishment and classification of the next scene model.

### Component analysis and extraction experiment

This paper simplifies the classification of the sample data and sorts out the corner space components of underground commercial streets from two aspects (spatial scale and spatial form) to obtain its main components.

#### Elements of spatial form

The symbiotic relationship between the corners, node space, and streets is affected by the street space scale. Usually, the corner appears at the turn or intersection of the street, so its local scale will change. The components of the corner scale mainly include the height, length, and width of the shop bay as well as the shop opening height, and shop depth (Hong and Zhongwei, 2016). According to the survey, the street height of underground commercial streets is between 3.0 and 4.0 m, the street length is between 55 and 80 m, and the street width is between 3.5 and 6.3 m. The average size of shop openings is 6.1 m, with the sizes ranging from 3.3 to 8.1 m. The shop heights are basically between 3.0 and 4.0 m, and shop depths vary from 6.0 to 10.0 m. According to the survey data calculations, the aspect ratio of underground commercial streets is between 0.8 and 2.3.

#### Components of space form elements

The corner space of underground commercial streets form various interfaces that enclose the corner space. The authors’ literature review and field investigations found that the corner space forms elements, which mainly include the corner mode, corner angle and intersection pattern. Thus, corner angle and corner mode are important factors that directly constitute corner space (Admiraal and Cornaro, 2016; Volchko et al., 2019). According to the investigation data, underground commercial street corner space angles can be categorized into acute angle, right angle and obtuse angle, accounting for 6.7%, 16.7%, and 76.7%, respectively. The underground street corner types include sharp corners, streamlined corners, cut corners, and shaded corners, accounting for 23.3%, 30%, 26.7%, and 20%, respectively (Guo and Zhou, 2004; Gu et al., 2019).

### Component extraction experiment

Based on the research samples and data collation, the main elements of the underground commercial street corner space form were extracted to establish typical scene models and to simulate real scenes *via* virtual reality technology.

#### Typical scene model establishment and experiment design

##### Typical scene model establishment

Based on the sample data, the authors found the zigzag street in L-shaped intersection spaces to be the most common; thus, an L-shaped street represents the typical scene for the model experiment. First, simplified modeling was carried out according to the corner space survey data of the underground commercial streets. Virtual reality technology was used to launch an immersive experience with the model, and experiments were carried out with psychological methods to collect questionnaire data. Then, with the help of the semantic differential method, the constituent elements of the underground commercial street’s corner space were screened and sorted out through cognitive evaluation, and the ratings were based on the subjects’ reactions to the scene models of different spatial forms in the scene.

##### Experiment design

The experiment was carried out in a laboratory with immersive virtual reality technology. The experiment hardware includes a computer host, display, an Oculus Rift virtual reality helmet, four space locators and a virtual joystick. The software is a graphics workstation with a GeForce GTX TITAN X graphics card and an ErgoLAB man–machine environment synchronization platform system. The experiment site is a confined 9 m × 5 m × 3 m, area with good privacy and light protection, free of interference from external factors. Subjects of all ages (22 males and 18 females) volunteered to participate in the experiment. According to the collection and collation of basic information, 82.5% of the subjects in the experiment often visited underground commercial streets and had normal psychological perceptions.

Subjects gave feedback on their spatial experience of subjective feelings while experiencing different virtual scenes. The experiment was mainly divided into two stages. In the first stage, the five-level Likert scale (very poor, generally poor, ambiguous, generally good, very good) was used to record the psychological cognition of the subjects (Guo and Zhou, 2004). Based on our literature review, answers to the Likert-scale questions, discussions with experts, and answers to on-the-spot questions, the corresponding descriptive words were selected to describe the psychological perception of the subjects’ scene model, as shown in Table 1 (Gu et al., 2019). Each group of descriptors in the questionnaire is divided into five levels according to the evaluation scale (very poor, generally poor, ambiguous, generally good, very good). The subjects are required to choose the appropriate level from the five levels in accordance with their psychological perception. In the second stage, the subjects are required to sort the spatial elements according to their importance. In the third stage of this of underground commercial street research, the main components that affect the psychological perception of the spatial form are sorted out based on the overall ranking weight.

**Table 1:** Description of the questionnaire.

Type	Questionnaire descriptor		
Spatial scale	Degree of spaciousness	Openness	Sense of scale
Spatial atmosphere	Interest	Relaxation	Comfort level
Spatial form	Abundance	Logic	Coordination

#### Experiment tasks and data collation

During the experiment, the subjects can roam and experience in different street models for 3–5 min with the help of operating handles, and the experimenter can guide the subjects to travel in space. After completing the tour, the subjects scored the spatial scale, spatial atmosphere and spatial form of each scene without departing from the experiment scene. Then the spatial elements of each scene were ranked based on weight and the experimenters recorded the questionnaire data of each scene.

A total of 41 questionnaires were collected in this experiment, including 40 valid questionnaires and one invalid questionnaire, with an effective rate of 97.6%. Through data processing, the score table obtains the cognitive evaluation of the scene space and the comprehensive ranking score chart is revealed as shown in Table 2. Sorting out the questionnaire data, we get the comprehensive ranking score of the scene space of the underground space, from high to low: corner mode, corner angle, aspect ratio, length, shop bay, height, width.

**Table 2:** Scores of cognitive evaluation of scene space.

Scene model	No.	Height (m)	Width (m)	Length (m)	Shop bay	Aspect ratio	Corner angle	corner mode	Space experience	Degree of satisfaction
Scene 1	S5	3.4	5.4	60	6	1.8	Blunt angle	Streamlined corner	0.67	1.1
Scene 2	S1	5.1	7.5	80	8	1.47	Blunt angle	Cut corner	0.66	0.9
Scene 3	S4	3.3	3.5	35	6	1.1	Blunt angle	Concave corner	0.43	0.6
Scene 4	S3	3	4	50	4	1.33	Acute angle	Streamlined corner	0.17	0.3
Scene 5	S2	3	3.6	50	3	1.2	Right angle	Cut corner	0.27	0.3

### Experiment data analysis

#### Reliability analysis of experiment data

Table 3 shows the results of statistical and reliability analysis of the experiment data processed by SPSS 24.0. The normalized Cronbach *α* coefficient value of each spatial cognitive data in the scene is 0.866 (the closer the *α* coefficient value is to 1, the higher its reliability). Table 3 shows the data collected as having successfully passed the reliability and validity tests. The data has high research value and can be used for the next correlation analysis.

**Table 3:** Total factor reliability coefficients.

Description	Corrected items total correlation (CITC)	Α coefficient of deleted item	Normalized Cronbach *α* coefficient
Spaciousness	0.586	0.852	0.866
Openness	0.655	0.846	
Sense of scale	0.519	0.858	
Interest	0.684	0.844	
Relaxation	0.715	0.842	
Comfort	0.668	0.846	
Richness	0.54	0.858	
Logic	0.264	0.879	
Coordination	0.678	0.845	
Overall satisfaction	0.549	0.856	

Tables 3–7 list the height and height-width ratio of streets at the corner space forms, which have strong correlation with psychological perception, such as spaciousness, sense of scale and logic. There is a certain correlation between street length and psychological perception. Compared with the effects of street scale on spatial cognition, the effects of the street corner mode and corner angle is more prominent. There is a strong positive correlation between the street corner mode’s width-height ratio, the corner angle, and the overall satisfaction of space. Conversely, the formation of shops on the side streets has had little impact on the corner space.

**Table 4:** Space cognition correlation analysis.

Variables	Street width	Street height	Street length	Corner mode	Aspect ratio	Shop opening	Spaciousness	Openness	Sense of scale	Interest	Relaxation	Comfort	Richness	Logic	Coordination	Satisfaction
Spaciousness	0.914^*^	0.815	0.787^**^	0.564^**^	0.914^*^	0.871	1									
Openness	0.489	0.819	0.329	−0.55	0.957^*^	0.393	0.695^**^	1								
Sense of scale	−0.899^*^	0.947^**^	−0.960^**^	0.853^**^	0.445^**^	−0.941^*^	0.259	0.466^**^	1							
Interest	−0.13	−0.069	−0.263	−0.163	0.146	−0.226	0.209	0.496^**^	0.432^**^	1						
Relaxation	0.591	0.088	0.592	0.687^**^	0.919^*^	0.671	0.469^**^	0.553^**^	0.512^**^	0.519^**^	1					
Comfort	0.413	0.108	0.323	0.441	0.954^**^	0.407	0.288	0.413^**^	0.400^*^	0.611^**^	0.630^**^	1				
Richness	−0.187	−0.285	−0.302	0.845^**^	0.045	0.519^*^	0.16	0.254	0.168	0.557^**^	0.399^*^	0.460^**^	1			
Logic	0.834	0.967^**^	0.758	−0.436	0.204	0.736	0.469^**^	0.306	0.184	0.175	0.218	0.258	0.086	1		
Coordination	0.759	0.399^*^	−0.084	0.657^**^	0.865^**^	0.975^**^	0.391^*^	0.369^*^	0.266	0.351^*^	0.449^**^	0.645^**^	0.493^**^	0.395^*^	1	
Satisfaction	−0.092	−0.408	−0.126	0.725^**^	0.763^**^	0.016	0.072	0.118	0.09	0.209	0.679	0.597	0.584	0.448	0.427	1

* Indicates significant correlation at the level of 0.05 (two-sided test).

** indicates significant correlation at the level of 0.01 (two-sided test).

**Table 5:** Collection of cognitive adjective pairs in space.

No	Adjectives	No.	Adjectives	No.	Adjectives
1	Broad-narrow	7	Boring-interesting	13	Complex-concise
2	Towering-low	8	Uneasy-secure	14	Unbalanced-coordinated
3	Open-closed	9	Uncomfortable-comfortable	15	Monotonous-various
4	Longer-shorter	10	Depressing-relaxing	16	Ugly-beautiful
5	Smaller-larger	11	Alienated-cordial	17	Closed-unobstructed
6	Intermittent-continuous	12	Angular-streamlined	18	Ordinary-peculiar

**Table 6:** Quantifiers of spatial psychological cognitive evaluation.

No.	Evaluating indicator	Adjectives	No.	Evaluation indicator	Adjectives
1	Interest	Boring-interesting	5	Relaxation	Depressing-relaxing
2	Openness	Broad-narrow	6	Continuity	Intermittent-continuous
3	Aesthetics	Ordinary-peculiar	7	Kindness	Alienated-cordial
4	Comfort	Uncomfortable-comfortable	8	Overall rating	Lower-Higher

**Table 7:** Item reliability coefficients.

Selections	Interest	Relaxation	Comfort	Openness	Kindness	Aesthetics	Continuity	Overall rating
Cronbach *α*	0.77	0.73	0.78	0.703	0.768	0.775	0.81	0.767
Cronbach *α* based on standardized item	0.782	0.733	0.771	0.704	0.762	0.778	0.814	0.768
Total Cronbach *α*	0.961							

#### Correlation analysis of experiment data

Correlation analysis is used to analyze the correlation between the components of the corner space and psychological perception. The Pearson correlation coefficient was employed to indicate the strength of the correlations, and the five-level Likert scale (very poor, average poor, ambiguous, average good, very good) was adopted. The analysis results are shown in Table 4.

### Pre-experiment conclusion

Conclusions concerning the progress of pre-experiments, factor analyses and a comprehensive evaluation on the corner space model scenes of five different types of underground commercial streets follow:

1. The cognitive experience of corner space has three components: (1) a street width-height ratio, (2) a corner mode, and (3) a corner angle. All components have a strong correlation with psychological perception; however, the components of street length, opening height, shop width, and shop depth have weak correlations. One of the stronger correlation factors is the ratio of width to height, which can directly affect the spatial scale cognition of underground commercial streets and has an important impact on the psychological perception of subjects. The angle and spatial processing techniques affect the space atmosphere cognition and morphological cognition of the corner space, which has a strong correlation with the pedestrian’s spatial comfort level experience.
2. In the contrast experiment of the whole experiment scene, the aspect ratio of the space in the underground commercial street is directly related to the cognition of all spatial forms. This is because unlike the above-ground commercial street, the underground commercial street has a stronger sense of enclosure, and the aspect ratio directly affects the psychological cognition of the subjects in the experiment stage.
3. Straight streets have a good sense of direction and bring good traffic convenience, but for underground commercial streets, the traffic convenience is not an important factor when testing their space experience. In the process of investigation, we found that most subjects think that straight streets are monotonous and boring, which makes it difficult to effectively convince pedestrians to stay. However, the appearance of corner space can change users’ psychological perception to a certain extent, and the angle of corner space can arouse users’ interest in space to a large extent.
4. Generally speaking, the subjects’ evaluation of various street scenes in the experiment is basically consistent with on-site investigation and evaluation, which indicates that the simulated pre-experiment has a similarity to existing street scenes, giving it a practical significance. At the same time, the results of data analysis also show that virtual reality technology is reliable in simulating the psychological perception of underground commercial streets, thereby providing a credible basis for the next experiment.

## Experimental analysis of the correlation between the elements of underground commercial streets’ corner space and psychological perception based on virtual reality technology

Based on the above data and pre-experimental analysis, three factors clearly affected the psychological perception of the corner space on underground commercial streets, the width-height ratio, the corner mode, and the corner angle. The following section summarizes the correlation between these three factors and psychological perception.

### Experiment on the correlation between the elements and psychological perception

In the relevance experiment, different corner space spatial shape models were constructed to accommodate the underground commercial streets’ need for classification and an orthogonal analysis of the elements. The semantic difference method was applied to project the psychological perception of the subjects and to make a quantitative analysis able to draw the relevant conclusions associated with the aspect ratio, corner mode and corner angle.

#### Model construction of virtual scenes

According to the pre-experiment conclusions regarding the corner space cognitive experience, the three indexes (the street height-width ratio, corner mode and corner angle) have a direct impact on the underground commercial street corner space cognition. Therefore, this experiment adopted the three basic independent variables to serve as the influencing factors of psychological cognition.

According to the sorting and analysis of previous research data, the high-frequency values of width are 4, 5, and 6.3 m, and the values of height are 3, 3.5, and 4 m, respectively. The aspect ratio of the scene formed by the orthogonal experiment of width and height is between 1 and 2.1, which accords with the distribution of the common street space aspect ratio in the research data. The high frequency values of the corners are 45°, 90°, 120°, and 150°. Four high frequency types of corner layout patterns were used in this research: cutting corner, sharp corner, streamline corner, and concave corner. The experiment scenes are composed of four types of design factors, each with their own level. The orthogonal analysis method is used to optimize the design factors and levels, forming 16 representative experiment scenes, as shown in Supplementary Appendix A.

In Supplementary Appendix A, a broken-line street is formed by two intersecting streets selected for each scene. The total length of the street is 70 m, which is common in this type of research. The ceiling in the scene is all made of wood-colored aluminum gusset, and the data for each shop shows it to have a 6-m width and an 8-m depth, with the specific shape adjusted locally according to the street. White cement was used on the wall of the shop; moreover, glass doors and the same signboard were used in all scenes. Beige porcelain tiles were used on the ground, and the illumination of a 700 lumen light inside the scene was generally suitable for underground streets in China. Other unspecified factors in the scene were treated in a unified manner.

#### Experiment and questionnaire design

This experiment was completed in the immersive equipment platform of a VR laboratory. Seven-level Likert scales (super poor, very poor, poor, fair, good, very good, and super good) were adopted, and 0 was set as the evaluation midpoint. Each pair of semantic words from −3 to 3 corresponded to seven scoring standards. Participants were required to score 16 corner space scenes while experiencing them. The scoring indexes were divided into seven spatial sub-scores and one spatial overall score, as shown in Table 5. In this experiment, 18 spatial cognitive adjectives were collected by using a semantic scale vocabulary screening and spatial cognition literature. Participants were asked to screen out seven adjectives (see Table 6) as the evaluation indexes of spatial psychological perception in this study. A total of 53 volunteers (28 males and 25 females, between 17 and 28 years old) were recruited for this study. This experiment had an effective rate of 86.8%, with 46 valid questionnaires and 7 invalid ones.

### Analysis of the relationship between components and psychological perception

After the experiment, 46 valid data were analyzed comprehensively by SPSS 24.0, including reliability & validity, correlation, comprehensive ranking, scatter plot, and stay activity.

#### Reliability analysis and validity test

##### Reliability analysis

Originating from psychological measurements, reliability analysis is generally used to test the reliability of questionnaire results. This reliability analysis was conducted using the questionnaire data of the eight variables and the total items in this experiment. The results are shown in Table 7.

Table 7 shows the Cronbach *α* coefficient of partial reliability for eight variables in the questionnaire at ≥0.7, and that the total Cronbach *α* is 0.961. This indicates that the experiment data are reliable and the scale results are authentic.

##### Validity test

The KMO test was used to check the correlation and partial correlation between variables. Bartlett’s Test of Sphericity was employed to test the correlation between variables in the correlation matrix, that is, whether each variable was independent or not.

Sorting out the experimental data to get the KMO test coefficient is 0.787, indicating a strong correlation between variables. Bartlett’s Test of Sphericity shows the significance is infinitely close to 0, implying good validity.

#### Correlation analysis

The normality test showed that collected scale data conform to normal distribution. Thus, the Pearson correlation analysis method can be used for correlation analysis and the results are shown in Table 8.

**Table 8:** Correlation analysis of cognitive quantity in each scene dimension.

Variables	Aspect ratio	Corner angle	Interest	Relaxation	Comfort	Openness	Kindness	Aesthetics	Continuity	Overall rating
Aspect ratio	1									
									
Corner angle	0.003	1								
	0.991									
Interest	0.394	0.344	1							
	0.131	0.192								
Relaxation	0.563^*^	0.531^*^	0.786^**^	1						
	0.023	0.034	0							
Comfort	0.522^*^	0.568^*^	0.746^**^	0.981^**^	1					
	0.038	0.022	0.001	0						
Openness	0.680^**^	0.388	0.717^**^	0.919^**^	0.890^**^	1				
	0.004	0.137	0.002	0	0					
Kindness	0.534^*^	0.508^*^	0.706^**^	0.923^**^	0.954^**^	0.887^**^	1			
	0.033	0.045	0.002	0	0	0				
Aesthetics	0.541^*^	0.451	0.849^**^	0.966^**^	0.953^**^	0.903^**^	0.937^**^	1		
	0.03	0.08	0	0	0	0	0			
Continuity	0.337	0.804^**^	0.639^**^	0.832^**^	0.864^**^	0.658^**^	0.844^**^	0.810^**^	1	
	0.202	0	0.008	0	0	0.006	0	0		
Overall rating	0.513^*^	0.565^*^	0.754^**^	0.946^**^	0.935^**^	0.838^**^	0.933^**^	0.945^**^	0.900^**^	1
	0.042	0.022	0.001	0	0	0	0	0	0	

* Indicates correlation significance at 0.05 level (two-sided test).

** indicates correlation significance at 0.01 level (two-sided test).

Table 8 shows the significance values (*p*) among eight psychological perceptions, such as corner space interest. All are <0.005, which indicates that there is a significant correlation among them. The data analysis results show that: (1) The closest correlation occurs between the relaxation of a space and its comfort and kindness, as the Pearson correlation coefficients are 0.905 and 0.880, respectively. This means a relaxing atmosphere can make users feel comfortable and effectively alleviate the negative impact of underground space. (2) The kindness of a space is significantly correlated with continuity and aesthetics, as the correlation coefficients are 0.844 and 0.937, indicating that users tend to enjoy continuous and aesthetic space interfaces. (3) Interest, relaxation, and the comfort degree of space have correlation coefficients of 0.849, 0.996, and 0.953, respectively. As factors of space atmosphere perception, interest, relaxation, and comfort degrees are all positively correlated with aesthetics, indicating that aesthetic space can create a more relaxing and comfortable psychological environment, thereby reducing users’ negative resistance. (4) The overall score of the scene is correlated with each psychological perception index, and it is highly correlated with the aesthetics, relaxation, kindness, and comfort of the corner space. (5) The ratio of street width to height in a corner space is positively correlated with the overall evaluation, with a correlation coefficient of 0.513. (6) A strong correlation exists between the angle of the corner space and continuity, with the correlation coefficient being 0.804. The angle is positively correlated with comfort and satisfaction with correlation coefficients of 0.568 and 0.565, respectively.

#### Comprehensive ranking analysis

According to our basic analysis and correlation analysis of the eight psychological perceptions of the corner space, a comprehensive ranking analysis can be made for each scene, as shown in Table 9.

**Table 9:** Scene classification index and comprehensive scores.

No.	Interest	Relaxation	Comfort	Openness	Kindness	Aesthetics	Continuity	Overall rating	Comprehensive scores
S5	15	15	16	11	16	15	16	15	119
S7	16	16	14	16	11	16	13	16	118
S4	13	14	15	14	15	13	15	13	112
S13	12	12	11	13	13	14	10	14	99
S9	14	13	12	15	12	12	8	12	98
S14	10	11	13	12	14	11	12	8	91
S10	11	8	10	7	10	10	11	10	77
S12	8	10	9	10	9	9	7	11	73
S1	5	9	7	4	5	6	14	9	59
S8	9	6	5	8	6	8	5	4	51
S15	4	5	6	6	7	7	6	7	48
S2	1	7	8	9	8	4	4	6	47
S11	6	4	4	5	3	5	3	3	33
S6	3	3	3	3	4	3	9	5	33
S16	7	2	2	2	2	2	2	2	21
S3	2	1	1	1	1	1	1	1	9

As can be seen from Table 9, the comprehensive ranking of scenes is basically consistent with that of individual spatial elements. Among them, S5, S7, and S4 are at the top of eight indexes and can be taken as the first tier. The comprehensive scores of S13, S9, S14, S10, S12, and S11 are between 59 and 99; thus, their evaluation scores are ranked 4–9 in the middle stage, so they belong to the second tier, S8. The comprehensive scores and each spatial element of S15, S2, S11, S6, S16, and S3 are all at the rear of the ranking and are given an S3 index, which is classified as the third tier.

#### Scatter point analysis

**Figure 2:**
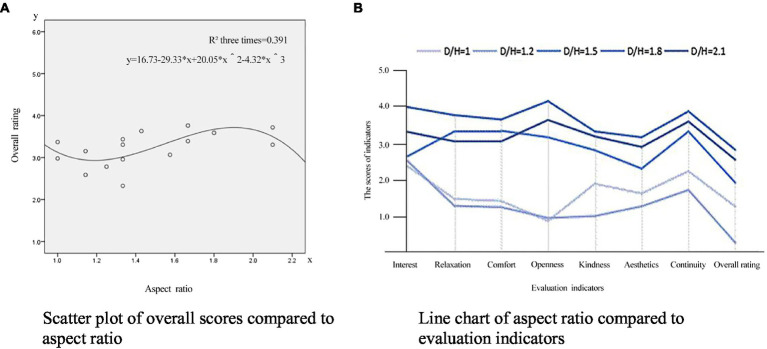
Scatter analysis of the aspect ratio against the overall rating. **(A)** Scatter plot of overall scores compared to aspect ratio. **(B)** Line chart of aspect ratio compared to evaluation indicators.

**Figure 3:**
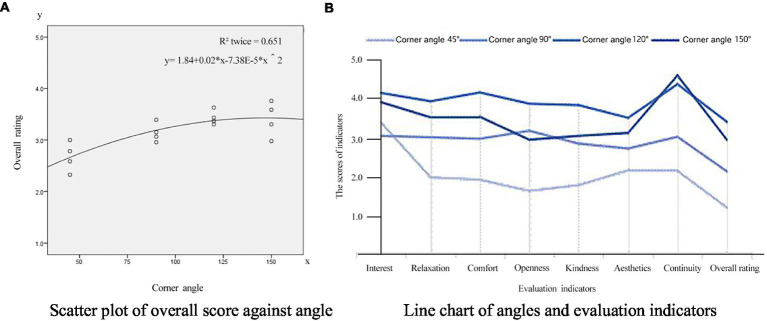
Analysis of corner angle compared to overall rating. **(A)** Scatter plot of overall score against angle. **(B)** Line chart of angles and evaluation indicators.

**Figure 4:**
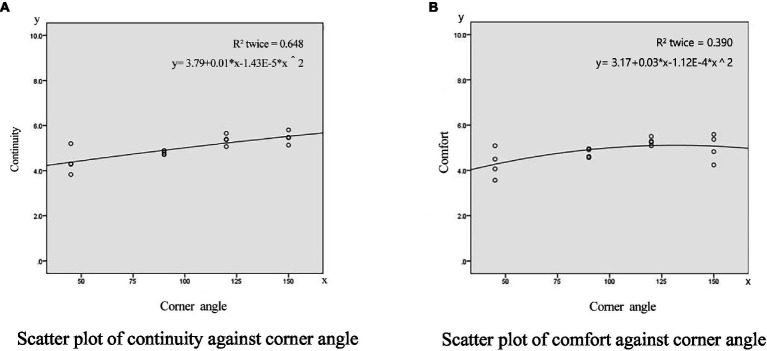
The scatter analysis of the spatial corner angle. **(A)** Scatter plot of continuity against corner angle. **(B)** Scatter plot of comfort against corner angle.

Scatter plots can show a trend of correlation between dependent and independent variables according to the distribution and density of data points. From the above correlation analysis, the aspect ratio, angle, and layout patterns are highly correlated with the overall rating of the corner space. Therefore, according to the experimental data shown in Figures 2A, 3A, 4, the scatter diagram covering the overall rating and the aspect ratio, corner angle, and layout patterns were drawn to reflect the relationship. At the same time, the scatter diagram was used to draw the fitting curve, and the regression equation was obtained to reflect the correlation trend between these design elements and the overall rating of the angular space.

##### Scatter analysis of the aspect ratio against the overall rating

Figure 2A shows a scatter analysis between an underground commercial streets’ corner space aspect ratio and the overall rating. In this case, the corner space aspect ratio of the experiment scenes is between 1.0 and 2.1. With the increase of the aspect ratio, the overall score of the space shows a curve change. When the aspect ratio of a corner space is between 1.1 and 1.3, the overall rating is lower. When the aspect ratio is between 1.5 and 1.8, the overall rating increases with the aspect ratio reaching the highest level at 1.8. When the aspect ratio is >2, the overall rating is negatively correlated with the aspect ratio, and the overall rating decreases accordingly. According to the fitting curve and Figure 2B (line chart of the classification index of streets with different aspect ratios), the psychological perception index score of a corner space is higher when the aspect ratio is between 1.5 and 1.8.

##### Analysis of corner angle compared to overall rating

The scatter analysis between the corner angle of underground commercial streets and overall rating are shown in Figure 3A. As the angle increased, the overall rating also gradually increased. The overall rating of the acute angle scene is obviously smaller than that of the obtuse angle scene, and when the angle is >125°, the overall rating of the space is basically flat. According to the Figure 3B line chart’s corner angle comparison to each evaluation indicator, when the corner angle of the scene is 120°, the scores of indicators (except for spatial continuity) and the overall rating are higher than that at the scene with a corner angle of 150°. This shows that the corner angle of underground commercial streets is closely related to the users’ cognitive evaluation, and the spatial experience is best when the corner angle is obtuse. However, when the corner angle is >150°, the spatial experience becomes less pleasant.

The scatter analysis of the spatial corner angle against continuity and comfort is shown in Figure 4. The corner angle has a strong correlation with continuity and comfort, and the spatial continuity becomes larger with the increase of the corner angle. When the angle of the corner approaches 180 degrees, the sense of the corner space is weakened, and the continuity evaluation is close to that of the streamlined street. Thus, the level of aesthetics and comfort gradually decreases.

##### Analysis of layout patterns against spatial evaluation

**Figure. 5:**
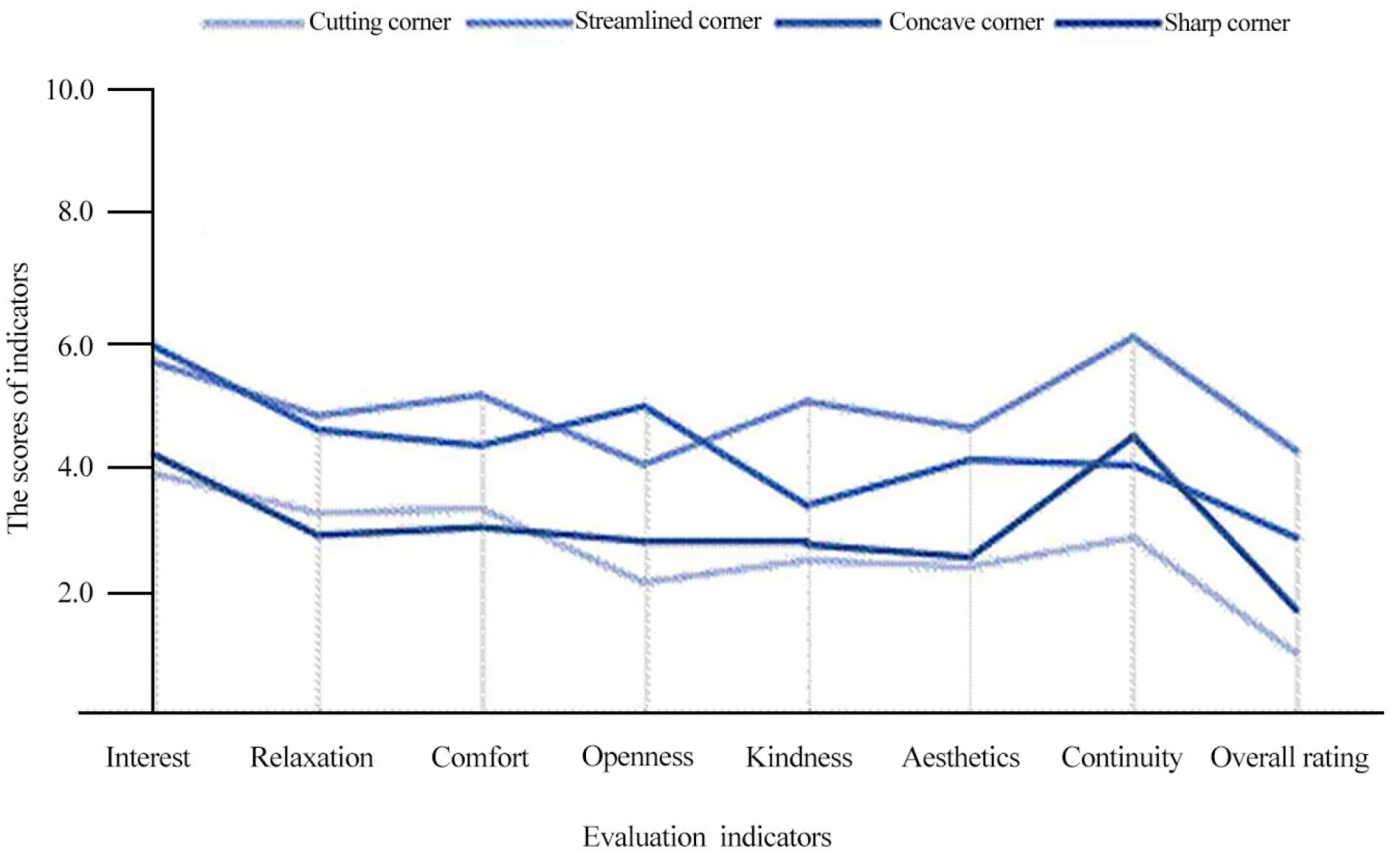
Line chart of layout patterns and evaluation indicators.

In this study, the layout patterns of corner space are divided into four types: sharp corner, streamlined corner, concave corner, and cut corner. Figure 5 shows the analysis of the corner space layout patterns against each evaluation indicator.

Figure 5 shows the evaluation index or corner space effectiveness based pm: (1) Among the four patterns of corner space, the scores of each of streamlined corner and concave corner indicator are obviously higher than those of a sharp corner. This indicates that the layout patterns of a corner space can affect the psychological perception of corner spaces on underground commercial streets to a certain extent. (2) The cut corner space score is high enough in spatial continuity, but the other indicators are average, which shows that a cut corner can strengthen the continuity of a corner space to a certain extent. In the streets with a strong commercial atmosphere, cut corner space can better continue the spatial atmosphere of the street and expand the influence of regional commerce. (3) Streamlined corners have the highest scores of all indicators, which suggests that this type of corner has positive effects thereby improving the users’ psychological cognition. (4) A concave corner, due to its concession on the corner interface, enhances the openness and interest of the space, but it weakens the continuity.

#### Analysis of stopping activities

In the stop-point selection experiment, subjects chose attractive or desirable places to stay in a simulated scene according to their self-cognition (personal preferences). The selection of the stopping point can be classified from two aspects — attractive streets or shops in the scene and streets or shops with comfortable space in the scene. Based on these two aspects (attractive appearance or space comfort). The participants chose the stopping points according to their cognitive evaluation. After the selection, the experiment’s monitor tallied the position stopping points and marked their location on the corresponding scene plans. After the 46 participants completed their marking, the stopping points of each scene are superimposed, and 736 stopping points were obtained in 16 scenes, as shown in Supplementary Appendix B. Analysis reveals that the participants’ selected corner stopping points were significantly affected by the layout patterns and the angles of corner space.

1. In the corner modes, the concave corner was more attractive to the test participants, followed by the streamlined corner, while the cut corner and the sharp corner were not as popular (i.e., less attractive). This may be related to the local variations of space and the aesthetics (attractiveness) of the interface form. The concave corner forms a wider local space due to the concession of the architectural interface, and the strong sense of enclosure makes the space appear more secure, i.e., more attractive to the test participants. However, streamlined corners often form an arc-shaped interface, thereby enhancing the aesthetics and making the test participants (virtual visitors in simulation set up) feel more comfortable.
2. In the corner angles, when the angle was acute, the street direction changed greatly, making the corner “tip” more prominent and attractive. Therefore, the shops in the corner were more likely to entice visitors to stay. However, when the angle became larger, the prominence of the “tip” gradually weakened. When the angle was >150°, the street tends to be straight, and the tip image in the corner space tends to be overlooked, causing the corner space to it lost its ability to make participants want to stay.
3. In the corner spaces among the shops on both sides of the street, test participants were more likely to stop at the outside shops compared with the shops on the inside. These corner space results indicate that the outside shops were more attractive than the inside shops to some extent. However, when the width of the street became smaller, the inside shops appeared to become more attractive to some degree.

## Conclusion

1. The aspect ratio is an important factor that affects the psychological perception of underground commercial streets’ corner space layout; its optimal value range is 1.5–1.8.
  According to the results of the authors’ correlation analysis, the aspect ratio (appearance preference compared to comfort preference), the corner space is positively correlated with the level of spaciousness, relaxation and aesthetics. This indicates that the aspect ratio of the corner space is an important factor affecting the psychological perception of all corner spaces in underground commercial streets. When the aspect ratio is between 1.5 and 1.8, the scores of all the corner space psychological perception indicators are higher, reaching the highest when the aspect ratio is 1.8. When the aspect ratio is >2, the spatial evaluation score becomes negatively correlated with the aspect ratio. With the increase of the aspect ratio, the overall corner space score decreases. Therefore, the corner space aspect ratio is crucial to the success of any corner interface design as it provides users with a better visual experience.

2. The corner angle directly affects the continuity of street corner space. When the angle is obtuse, the overall experience of the space is better, and the best angle should be between 120° and 150°. The corner angle of the underground commercial streets is closely related to the users’ psychological perception. When the angle is obtuse, the experience of the space is better, and the best corner angle should be between 120° and 150°. The angle of a corner space has a strong correlation with spatial continuity and comfort. As the angle increases, the spatial continuity gradually increases. When the corner angle approaches 180°, the corner space sense of depth is weakened, and the street space tends to be linear as its continuity evaluation approaches that of the streamlined streets, and the levels of aesthetics and comfort gradually decline. Therefore, this research suggests that the corner angle should be flexibly adjusted to meet various needs, especially when attempting to avoid negative emotions of users.
3. The patterns of corner space can effectively improve the psychological perception of users, especially streamlined and concave corners.

Streamlined corner spaces have a positive effect on the improvement of users’ psychological perception. They have smooth curves and can make the space change naturally, and users can accept the change of environment and direction. Concave corners have good evaluation on the openness and interest of space because of their concession on the corner interface, but they weaken the continuity of space to some extent. The sharp corner space and cut corner space make the transition of intersections more obvious, which is likely to be rejected by users. Therefore, it is suggested that streamlined corners and concave corners should be a priority in design to meet the psychological needs of users in the corner space of underground commercial streets.

4. Corner space can effectively attract people to shop, and the vitality of a corner space is related to the corner angle.

Different corner angles can affect users’ stay behavior in different ways. A concave corner space is more attractive to most users, followed by the streamlined corner; however, the cut corner, and sharp corner are the least attractive. Thus, corner shops can be more attractive. Among the shops on both sides of the street, those shops in the corner space and in the outside shops are more attractive than the inside ones, but when the street width becomes smaller, the inside shops may have their attractiveness enhanced to some degree. Therefore, it is suggested that shops can be properly located according to their function, to make full use of the different space attributes on both sides of the street.

## Future research and next steps

As an exploratory study of underground space morphology and psychological perception, this paper includes a relatively complete and reliable data collection, experimental methods, and comprehensive analysis. Furthermore, by analyzing the correlation between the constituent elements of underground space and psychological perception, this paper provides a reference for improving the design of corner space and lays a foundation for future in-depth research.

However, the paper still has a few shortcomings: First, the constituent elements of corner space used in the virtual scene model are all simplified, and the types of corner spaces are far fewer in reality, which partly affects the accuracy of the authors’ research conclusions. Second, in order to simplify the virtual scene model of underground corner space, the paper standardizes the elements such as shop facade structure, lighting and shop decorations of underground commercial streets. These elements however have a great impact on the psychological perception of the subjects and should be given a more in-depth discussion in future studies.

Future research can be carried out from the following two aspects: First, in addition to space form, the psychological perception of underground space is also closely related to environmental elements such as light and sound in space. Thus, these factors should be taken into account in future studies. Second, in the experiment studying the correlation between the constituent elements of underground space and psychological perception, electro-encephalogram technology can be used capture the subjects’ psychological perception. With the further assistance of computer analysis and machine learning algorithms, more accurate research results can be obtained.

## Data availability statement

The original contributions presented in the study are included in the article/Supplementary material, further inquiries can be directed to the corresponding author.

## Author contributions

TF: conceptualization, methodology, and software. YS: visualization and investigation. LS: supervision, software, and validation. ZS: reviewing and editing. All authors contributed to the article and approved the submitted version.

## Ethics statement

Ethical review and approval was not required for the study on human participants in accordance with the local legislation and institutional requirements. Written informed consent from the (patients/ participants OR patients/participants legal guardian/next of kin) was not required to participate in this study in accordance with the national legislation and the institutional requirements.

## Funding

This study has been financially supported by International Science and Technology Cooperation Fund of Jiangsu Collaborative Innovation Center for Building Energy Saving and Construction Technology (grant no. SJXTGJ2102), Graduate Innovation Program of China University of Mining and Technology (grant no. 2022WLJCRCZL299), and the Fundamental Research Funds for the Central Universities (grant no. 2020QN12).

## Conflict of interest

The authors declare that the research was conducted in the absence of any commercial or financial relationships that could be construed as a potential conflict of interest.

## Publisher’s note

All claims expressed in this article are solely those of the authors and do not necessarily represent those of their affiliated organizations, or those of the publisher, the editors and the reviewers. Any product that may be evaluated in this article, or claim that may be made by its manufacturer, is not guaranteed or endorsed by the publisher.

## References

[ref1] Admiraal H. Cornaro A. (2016). Why underground space should be included in urban planning policy-and how this will enhance an urban underground future. Tunn. Undergr. Space Technol. 55, 214–220. doi: 10.1016/j.tust.2015.11.013

[ref2] Bartel S. Janssen G. (2016). Underground spatial planning-perspectives and current research in Germany. Tunneling Underground Space Technol. 55, 112–117. doi: 10.1016/j.tust.2015.11.023

[ref3] Carmody J. Sterling R. (1993). Underground space design: a guide to subsurface utilization and design for people in underground spaces. Tunn. Undergr. Space Technol. 8, 390–391. doi: 10.1016/0886-7798(93)90036-U

[ref4] Gu Z. Osaragi T. Lu W. (2019). Simulating pedestrians’ spatio-temporal distribution in underground spaces. Sustain. Cities Soc. 48:101552. doi: 10.1016/j.scs.2019.101552

[ref5] Guo Q. K. Zhou J. (2004). Validity of different IRT models in Likert scale analysis. Psychol. Explor. 3, 67–70. doi: 10.3969/j.issn.1003-5184.2004.03.015

[ref6] Hong Y. Zhongwei S. (2016). Research on the evolution of underground space function and the development of design theory. J. Archit. 12, 77–82. doi: 10.3969/j.issn.0529-1399.2016.12.011

[ref7] Li J Wu W Jin Y Zhao R., Bian W. (2021). Research on environmental comfort and cognitive performance based on EEG+VR+LEC evaluation method in underground space. Build. Environ. 198:107886, ISSN doi: 10.1016/j.buildenv.2021.107886

[ref8] Mao Y. Y. Li W. C. Zhu M. M. Ding J. J. Zeng M. L. (2020). Research progress of the effect of underground space environment on behavior psychology in China. J. Hum. Settlements Western China 35, 58–66. doi: 10.13791/j.cnki.hsfwest.20200408

[ref9] Sun L. Tan W. Ren Y. Ji X. Wang Z. Li P. (2020). Research on visual comfort of underground commercial streets’ pavement in China on the basis of virtual simulation. Int. J. Pattern Recognit. Artif. Intell. 34:2050005. ISSN (print): 0218-0014 | ISSN (online): 1793-6381. doi: 10.1142/S0218001420500056

[ref10] Tong L. X. (2005). Underground Space and the Development of Urban Modernization. Beijing: China Construction Industry Publishing House.

[ref11] Volchko Y. Norrman J. Ericsson L. O. Nilsson K. L. Markstedt A. Oberg M.. (2019). Subsurface planning: towards a common understanding of the subsurface as a multifunctional resource. Land Use Policy 90:104316. doi: 10.1016/j.landusepol.2019.104316

[ref12] Wada Y. Sakugawa H. (1990). Psychological effects of working underground. Tunn. Undergr. Space Technol. 5, 33–37. doi: 10.1016/0886-7798(90)90060-W

[ref13] Yao G. Yuan T. Rui Y. Chen W. Duan Z. Sun L.. (2021). Research on the scale of pedestrian space in underground shopping streets based on VR experiment. J. Asian Archit. Build. Eng. 20, 138–153. doi: 10.1080/13467581.2020.1782215

