# Supplementary material for: Research on the correlation between component elements and psychological perception of the spatial form of underground commercial street corners based on virtual reality technology
Source: Front Psychol. 2022 Sep 6;13:950593. doi: 10.3389/fpsyg.2022.950593 (PMC9487714; doi:10.3389/fpsyg.2022.950593)

Appendix A Schematic diagram of experiment scenes

| Scene 1 | Model sketch  (partial) | 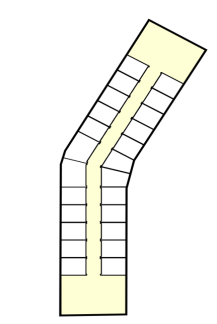 | | 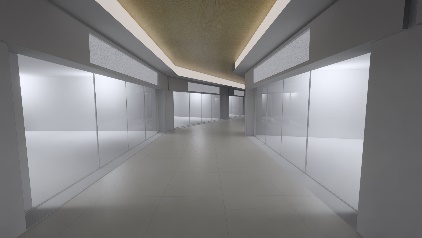 | | |
| --- | --- | --- | --- | --- | --- | --- |
|  | Factors | Width (m) | Height (m) | Corner angle  (degrees) | Aspect ratio | Layout pattern |
|  | Data | 4 | 3 | 150 | 1.3 | Sharp corner |
| Scene 2 | Model sketch  (partial) | 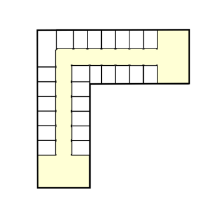 | | 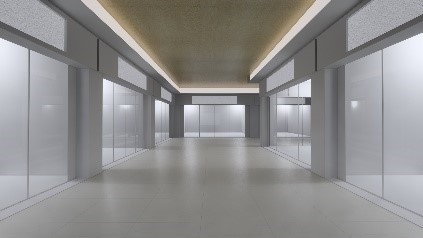 | | |
|  | Factors | Width (m) | Height (m) | Corner angle  (degrees) | Aspect ratio | Layout pattern |
|  | Data | 6.3 | 4 | 90 | 1.6 | Sharp corner |
| Scene 3 | Model sketch  (partial) | 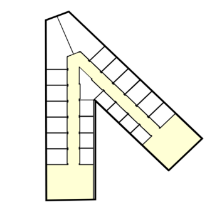 | | 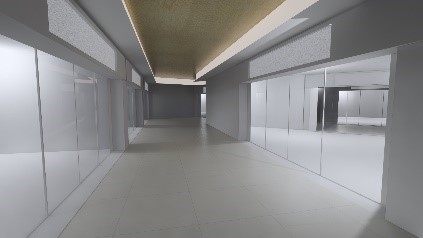 | | |
|  | Factors | Width (m) | Height (m) | Corner angle  (degrees) | Aspect ratio | Layout pattern |
|  | Data | 4 | 3 | 45 | 1.3 | Sharp corner |
| Scene 4 | Model sketch  (partial) | 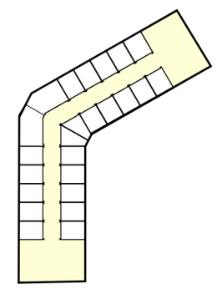 | | 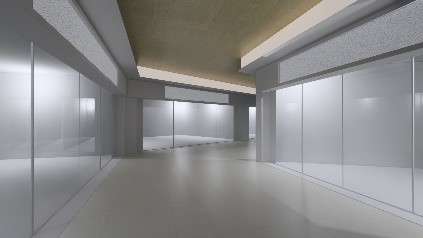 | | |
|  | Factors | Width (m) | Height (m) | Corner angle  (degrees) | Aspect ratio | Layout pattern |
|  | Data | 5 | 3.5 | 120 | 1.4 | Sharp corner |
| Scene 5 | Model sketch (partial) | 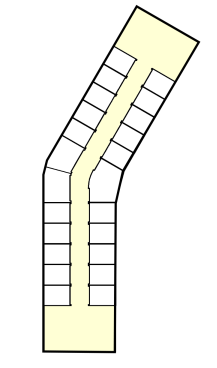 | | 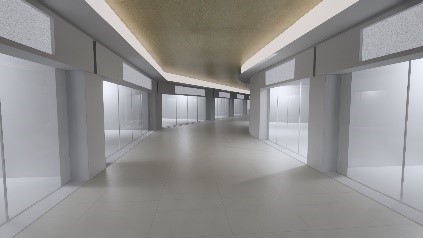 | | |
|  | Factors | Width (m) | Height (m) | Corner angle  (degrees) | Aspect ratio | Layout pattern |
|  | Data | 5 | 3 | 150 | 1.7 | Streamline corner |
| Scene 6 | Model sketch (partial) | 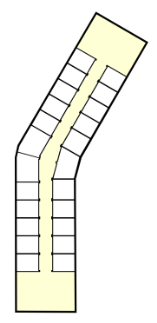 | | 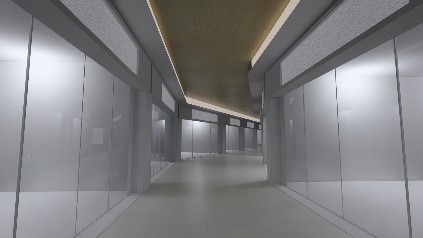 | | |
|  | Factors | Width (m) | Height (m) | Corner angle  (degrees) | Aspect ratio | Layout pattern |
|  | Data | 4 | 4 | 150 | 1 | Cutting corner |
| Scene 7 | Model sketch (partial) | 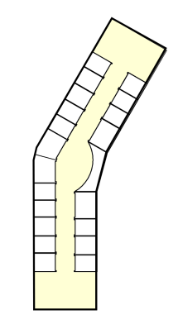 | | 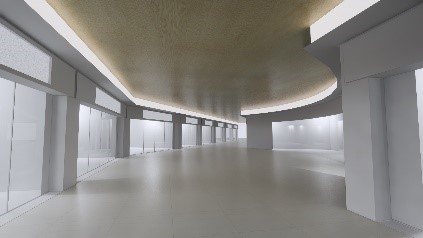 | | |
|  | Factors | Width (m) | Height (m) | Corner angle  (degrees) | Aspect ratio | Layout pattern |
|  | Data | 6.3 | 3.5 | 150 | 1.8 | Concave corner |
| Scene 8 | Model sketch (partial) | 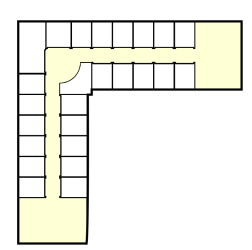 | | 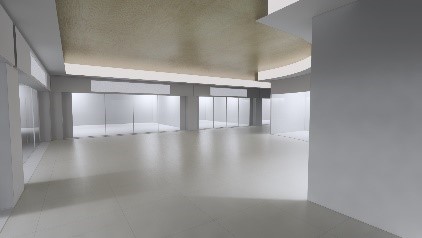 | | |
|  | Factors | Width (m) | Height (m) | Corner angle  (degrees) | Aspect ratio | Layout pattern |
|  | Data | 4 | 3 | 90 | 1.3 | Concave corner |
| Scene 9 | Model sketch (partial) | 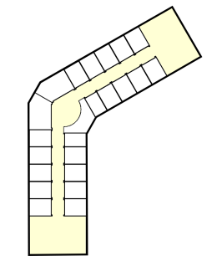 | | 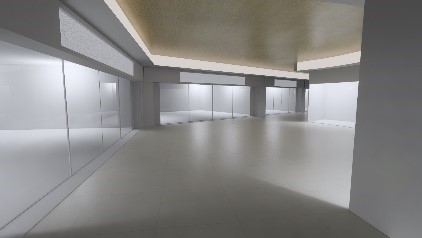 | | |
|  | Factors | Width (m) | Height (m) | Corner angle  (degrees) | Aspect ratio | Layout pattern |
|  | Data | 4 | 3 | 120 | 1.3 | Concave corner |
| Scene10 | Model sketch (partial) | 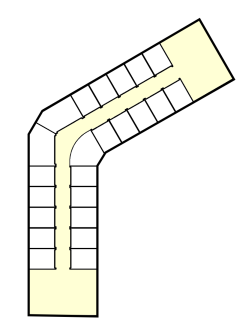 | | 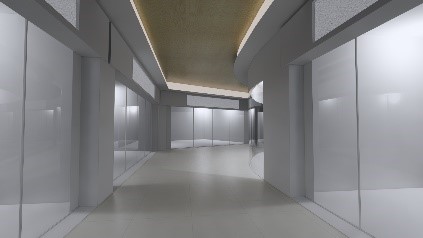 | | |
|  | Factors | Width (m) | Height (m) | Corner angle  (degrees) | Aspect ratio | Layout pattern |
|  | Data | 4 | 4 | 120 | 1.0 | Streamline corner |
| Scene 11 | Model sketch (partial) | 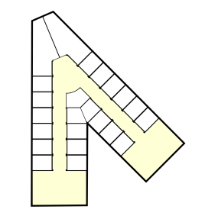 | | 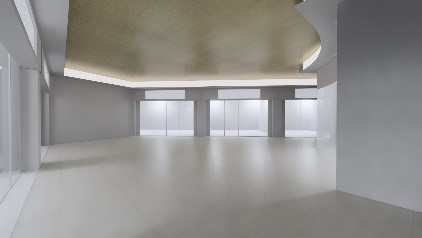 | | |
|  | Factors | Width (m) | Height (m) | Corner angle  (degrees) | Aspect ratio | Layout pattern |
|  | Data | 5 | 4 | 45 | 1.3 | Concave corner |
| Scene 12 | Model sketch (partial) | 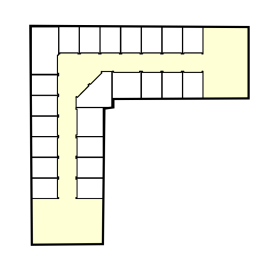 | | 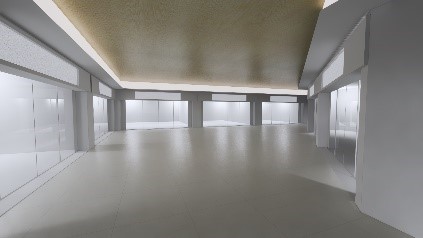 | | |
|  | Factors | Width (m) | Height (m) | Corner angle  (degrees) | Aspect ratio | Layout pattern |
|  | Data | 5 | 3 | 90 | 1.7 | Cutting corner |
| Scene 13 | Model sketch (partial) | 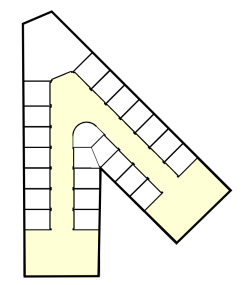 | | 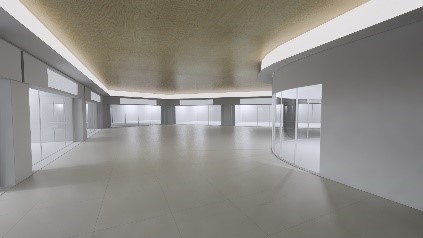 | | |
|  | Factors | Width (m) | Height (m) | Corner angle  (degrees) | Aspect ratio | Layout pattern |
|  | Data | 6.3 | 3 | 45 | 2.1 | Streamline corner |
| Scene 14 | Model sketch (partial) | 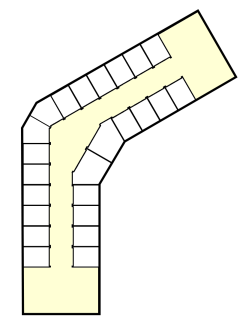 | | 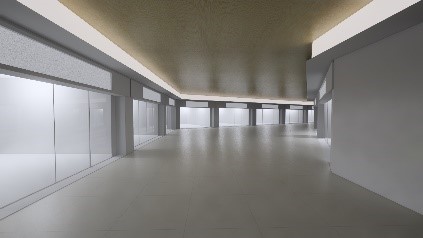 | | |
|  | Factors | Width (m) | Height (m) | Corner angle  (degrees) | Aspect ratio | Layout pattern |
|  | Data | 6.3 | 3 | 120 | 2.1 | Cutting corner |
| Scene 15 | Model sketch (partial) | 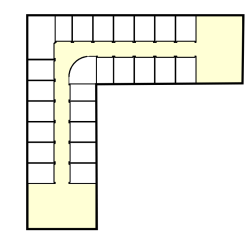 | | 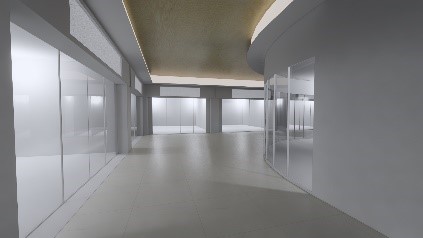 | | |
|  | Factors | Width (m) | Height (m) | Corner angle  (degrees) | Aspect ratio | Layout pattern |
|  | Data | 4 | 3.5 | 90 | 1.1 | Streamline corner |
| Scene 16 | Model sketch (partial) | 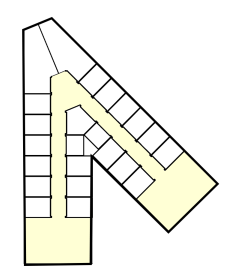 | | 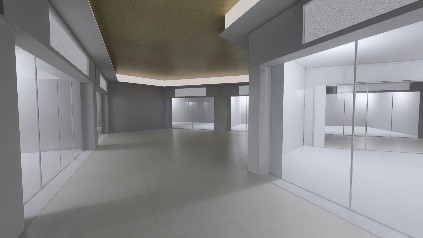 | | |
|  | Factors | Width (m) | Height (m) | Corner angle  (degrees) | Aspect ratio | Layout pattern |
|  | Data | 4 | 3.5 | 45 | 1.1 | Cutting corner |

Appendix B Statistical diagram of stopping points (represented by superimposed orange disks) in the corner space.
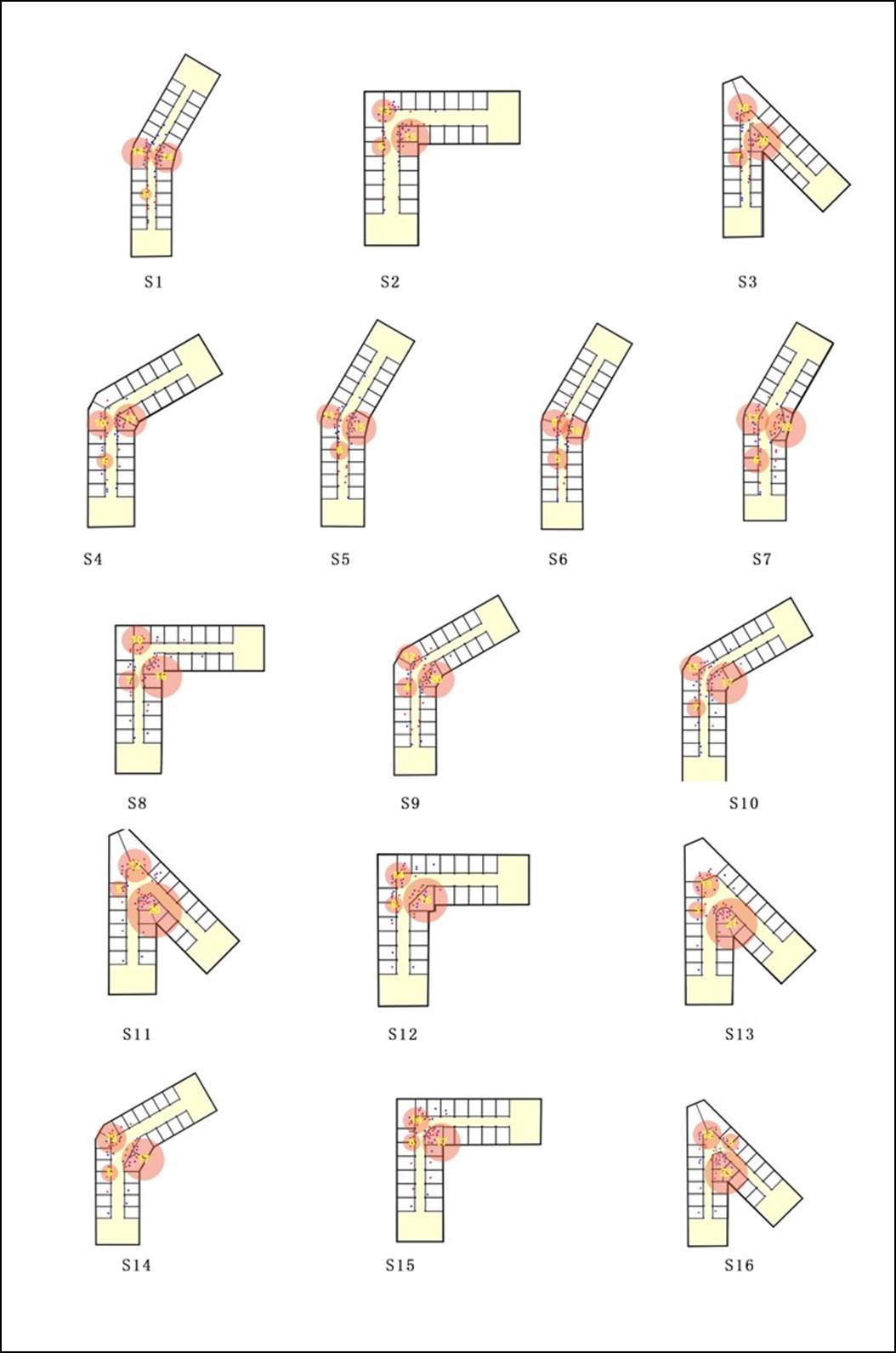

Supplement: Supplementary file 1 [file Data_Sheet_1.docx]
